# BMI-for-age *Z*-score is associated with prosthetic failure after endoprosthetic reconstruction in pediatric osteosarcoma

**DOI:** 10.1016/j.jbo.2026.100812

**Published:** 2026-10-31

**Authors:** Song Peng, Zhilong She, Jiahao Chen, Qiong Wu, Guangxin Zhou, Yicun Wang

**Affiliations:** a Department of Orthopedics, Jinling Clinical Medical College, Nanjing Medical University, Nanjing, Jiangsu, China; b Department of Orthopedics, Jinling Hospital, Medical School of Nanjing University, Nanjing, Jiangsu, China; c Department of Research Training, Jinling Hospital, Medical School of Nanjing University, Nanjing, Jiangsu, China

**Keywords:** Osteosarcoma, Pediatric, Endoprosthetic reconstruction, Limb salvage, BMI-for-age *Z*-score, Implant failure

## Abstract

**Background:**

Endoprosthetic reconstruction is a standard limb-salvage procedure for children and adolescents with osteosarcoma, but implant failure still limits long-term function. Body habitus influences outcome in adult arthroplasty, but the role of age-adjusted body mass index (BMI) in pediatric orthopaedic oncology remains unclear. We examined the association between BMI-for-age *Z*-scores and implant survival and whether it was nonlinear.

**Methods:**

At a tertiary center, we conducted a retrospective review of 239 consecutive patients with osteosarcoma treated with tumor resection and endoprosthetic reconstruction. BMI was converted to World Health Organization BMI-for-age *Z*-scores and ranked as low, normal, or high. The primary endpoint was implant failure. Implant survival was assessed using Fine-Gray competing-risk models, with death as a competing event. The dose-response relationship was assessed using restricted cubic splines. The Fine-Gray regression was used to analyze postoperative complications with sensitivity analysis for lower-extremity reconstruction.

**Results:**

Implant survival was lower in the low- and high-BMI groups than in the normal-BMI group. In Fine-Gray competing risk analysis, both low and high BMI were independently associated with implant failure (subdistribution hazard ratio [SHR] = 2.10, 95% confidence interval [CI]: 1.18–3.76; *P* = 0.012 and SHR = 2.28, 95% CI: 1.43–3.65; *P* = 0.001, respectively). Restricted cubic spline modeling revealed a significant U-shaped association between BMI-for-age *Z*-score and implant failure risk (nonlinear *P* = 0.022; overall association *P* = 0.001). Abnormal BMI was also associated with more postoperative complications, and this was consistent in the lower-extremity subgroup.

**Conclusions:**

In pediatric osteosarcoma, the BMI-for-age Z-score was significantly associated with implant failure, with a higher risk at both low and high values, thus demonstrating a U-shaped relationship. This supports the use of age-adjusted nutritional assessment for preoperative risk stratification in pediatric limb-salvage reconstruction.

## Highlights

• Extremes of BMI were associated with inferior outcomes after pediatric tumor endoprosthetic reconstruction.
• Pediatric BMI-for-age references may be more appropriate than adult BMI cutoffs in this population.
• Both underweight and overweight status may be clinically relevant in limb-salvage reconstruction.
• Preoperative BMI assessment may aid risk stratification and perioperative optimization.

## Graphical abstract

**Figure:**
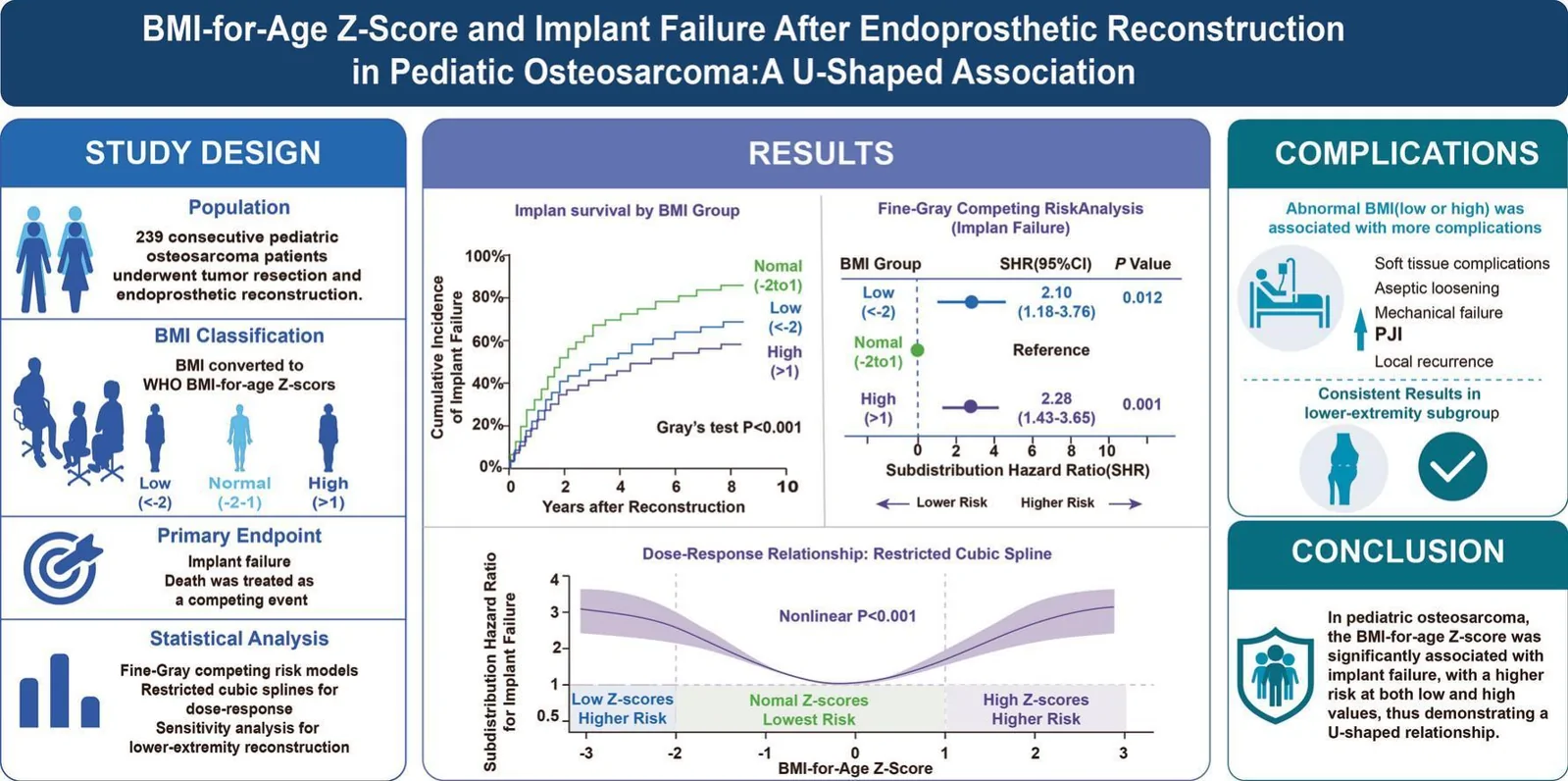

## 1 Introduction

Endoprosthetic reconstruction is now the standard limb-salvage treatment for children and adolescents with osteosarcoma, but implant failure still limits long-term durability and frequently results in early revision [1], [2]. This is particularly important for young individuals because the implant must last for decades, and many will require multiple revisions throughout their lives [2]. Most research on endoprosthetic failure has focused on tumor biology, surgical technique, and prosthetic design [2]. Patient factors that may be modifiable, particularly body composition and nutritional status, have received less attention [2], [3]. In adult arthroplasty, body mass index (BMI) is associated with perioperative complications, prosthetic loosening, and periprosthetic joint infection (PJI) [4], [5]. Adult data also exhibit higher risks of infection, aseptic loosening, and early revision at both low and high BMIs [4], [5], [6]. However, it remains unclear whether this information applies to children with osteosarcoma.

Children and adolescents with osteosarcoma differ from adult arthroplasty patients in terms of growth pattern, body composition, implant demand, and exposure to multi-agent chemotherapy [7]. Previous studies on BMI and endoprosthetic survival usually used mixed-age cohorts, yielding inconsistent results [8], [9]. A major problem is the reliance on adult BMI cutoffs instead of age- and sex-standardized BMI-for-age *Z*-scores, which are needed for nutritional assessment in growing children [10]. BMI-for-age Z-score does not directly measure lean mass or fat distribution, but it is a useful, validated marker of nutritional status in pediatric patients [11]. It is unclear whether it relates meaningfully to endoprosthetic survival after limb-salvage surgery for pediatric osteosarcoma. Previous research has had three common deficiencies: limited use of standardized pediatric BMI classification, insufficient reporting of specific failure modes, and failure to treat mortality as a competing risk in survival analysis [7], [12]. Additionally, low and high nutritional status may affect implant survival through different biologic and mechanical pathways, raising the possibility of a previously unexplored nonlinear association [5].

With this in mind, we conducted a retrospective cohort study to assess the impact of BMI-for-age *Z*-score on endoprosthetic survival in pediatric osteosarcoma patients treated with limb-salvage surgery and endoprosthetic reconstruction. We aimed to determine whether pediatric BMI categories independently predict implant failure and major postoperative complications and, if so, whether the association is linear or nonlinear.

## 2 Methods

### 2.1 Study population and methodology

We conducted a single-center retrospective cohort study at Jinling Hospital, an affiliated hospital of the Medical School, Nanjing University. Prior to data collection, the Institutional Review Board approved the study (No. 2025DZZDKT-039). Clinical data were obtained from a prospectively maintained electronic medical record database.

We included children and adolescents younger than 18 years with histologically confirmed osteosarcoma who underwent primary limb-salvage surgery with endoprosthetic reconstruction between January 2012 and December 2024. The inclusion criteria were as follows: Histopathologically confirmed osteosarcoma; tumor at the distal femur, proximal femur, proximal tibia, or proximal humerus; treatment with a tumor-type endoprosthetic replacement; and complete baseline and follow-up data. The exclusion criteria were as follows: Patients with follow-up <2 years without a study endpoint; death within 30 days after surgery; or missing height or weight data preventing BMI calculation. Fig. 1 illustrates patient selection.

**Fig. 1:**
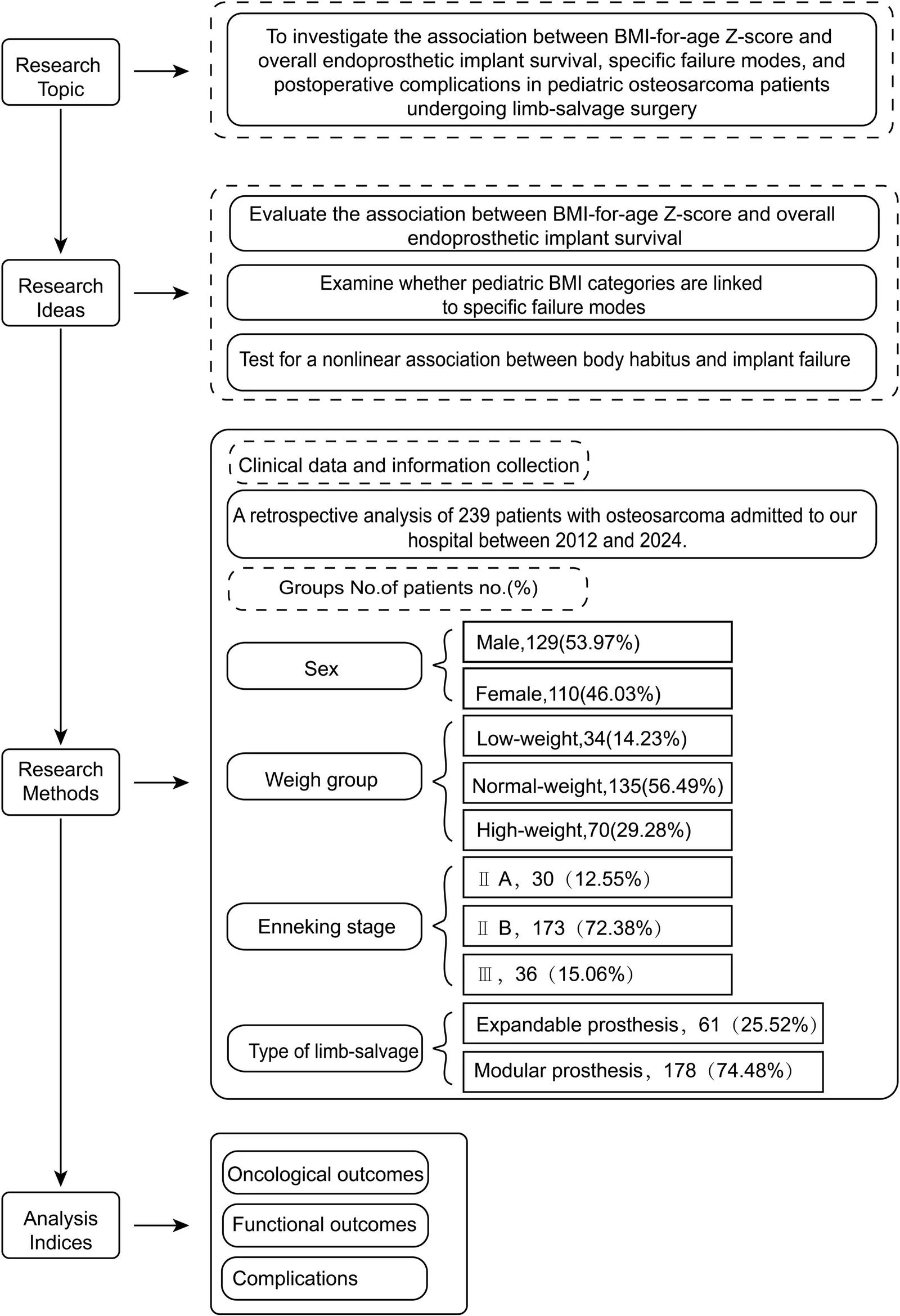
Schematic flow diagram of the study.

### 2.2 Outcome measures and definitions

The BMI was calculated as the weight of the patient in kilograms divided by height in meters squared. BMI-for-age *Z*-scores were calculated based on the World Health Organization (WHO) Child Growth Standards, and patients were categorized into three groups: Low BMI (Z-score < −2, thinness/undernutrition); normal BMI (−2 ≤ Z-score ≤ 1, healthy weight for age); high BMI (Z-score > 1, at risk of overweight by WHO definition). Tumor characteristics included anatomical location and Enneking stage. The type of prosthesis was recorded as modular or expandable.

The primary endpoint was implant failure, defined as unplanned secondary surgery on the original prosthesis, such as component removal, partial revision, or complete exchange. Planned lengthening procedures performed as part of the intended function of an expandable prosthesis were not classified as implant failure. When more than one unplanned secondary procedure was performed on the same prosthesis, only the first procedure was counted as the failure event, so that each patient contributed a single event to the time-to-event analysis; subsequent procedures on the same prosthesis were not counted as separate failures. Implant survival was measured from primary surgery to the first qualifying event, and patients without an event were censored at the final follow-up. Secondary endpoints were major postoperative complications by the Henderson classification: PJI, aseptic loosening, structural or mechanical prosthesis failure, local tumor recurrence, and soft tissue complications. In all analyses, all-cause mortality was considered a competing risk.

### 2.3 Statistical analysis

Continuous variables were compared using one-way analysis of variance or the Kruskal–Wallis H test, depending on distribution. Categorical variables were compared using the chi-square or Fisher's exact test. To identify independent predictors of implant failure, we used Fine-Gray competing risk regression with all-cause mortality as the competing event. The Aalen–Johansen method was used to estimate cumulative incidence functions (CIFs) for each postoperative complication, and 95% confidence intervals (CIs) were calculated by the Greenwood formula. In separate competing risk models, each complication was the primary endpoint, while death was treated as competing events. We also plotted stratified CIF curves according to the BMI *Z*-score subgroup.

To investigate the nonlinear dose-response relationship between continuous BMI Z-score and implant failure risk, we used restricted cubic spline regression with three knots at the 10th, 50th, and 90th percentiles of the Z-score distribution. Schoenfeld residual testing was used to check and confirm the proportional hazards assumption. All models were adjusted for age, sex, BMI stratification, tumor location, Enneking stage, and prosthesis type. A two-sided *P* < 0.05 was considered significant. The analyses were performed using R software (version 4.2.1) and Statistical Analysis System software (version 9.4).

## 3 Results

### 3.1 Baseline cohort characteristics

The final cohort consisted of 239 patients. During the follow-up period, 85 patients experienced implant failure, while 43 died without any implant failure. Additionally, 15 patients were excluded due to insufficient follow-up data. The median follow-up period was 8.2 years (range, 2.0–13.3). The mean age at surgery was 14.2 years (range, 5–18), with 46.03% being female. The mean age- and sex-adjusted BMI *Z*-score was 0.16 (range, −2.87 to 2.94).

According to BMI subgroup, 34 patients (14.23%) had low BMIs, 135 (56.49%) had normal BMIs, and 70 (29.29%) had high BMIs. The most common site was the distal femur (62.34%), followed by the proximal tibia (21.34%). Modular prostheses were used in 74.48% of cases, with expandable prostheses in 25.52%. Table 1 presents the baseline characteristics by BMI group.

**Table 1:** Baseline Characteristics of the Cohort (*n* = 239).

Variable	Low-weight	Normal-weight	High-weight	Total no. (%)
No. of patients	34	135	70	239
Sex				
Male	18	73	38	129
Female	16	62	32	110
Age of diagnosis, y				
Mean (std)	14.29(3.45)	13.90(3.31)	14.71(3.38)	14.19(3.35)
Median (range)	15(6–18)	14(5–18)	16(5–18)	15(5–18)
BMI				
<18.5	34	46	5	85
18.5–24	0	89	20	109
>24	0	0	45	45
*Z*-score				
Z < -2	34	0	0	34
-2 ≤ Z ≤ 1	0	135	0	135
Z > 1	0	0	70	70
Location of tumor				
Proximal humerus	2	6	3	11
Proximal femur	5	16	7	28
Distal femur	19	81	50	150
Proximal tibia	8	32	10	50
Enneking stage				
II A	3	17	10	30
II B	23	102	48	173
III	8	16	12	36
Type of limb-salvage				
Expandable prosthesis	10	37	14	61
Modular prosthesis	24	98	56	178
Survival status				
Survive	22	88	43	153
Deaths	12	47	27	86
Prosthetic Status				
Prosthetic Survival	17	103	34	154
Prosthetic Failure	17	32	36	85
Follows-up time, y				
Mean	8.2	8.1	8.3	8.2
Median (range)	8.1(2–12.8)	8.0(2–13.3)	8.3(2–13.1)	8.1(2–13.3)

Data are presented as mean (SD), median (range), or N (%).

### 3.2 Implant survival and nonlinear association

Implant failure occurred in 85 patients (35.56%). In the entire cohort, the 5-year cumulative incidence of failure was 34.88% (95% CI: 24.63%–45.14%), rising to 50.11% (95% CI: 40.55%–59.68%) at 10 years (Fig. 2). There were significant subgroup differences when stratified by BMI *Z*-score (Gray's test, *P* = 0.004). At 5 years, cumulative incidence was 44.64% (95% CI: 25.16%–64.13%) in the low-BMI group, 25.43% (95% CI: 12.98%–37.88%) in the normal-BMI group, and 47.12% (95% CI: 32.85%–61.39%) in the high-BMI group. The 10-year estimates were 55.49% (95% CI: 37.40%–73.58%), 38.72% (95% CI: 25.84%–51.59%), and 66.44% (95% CI: 54.18%–78.70%), respectively (Fig. 3).

**Fig. 2:**
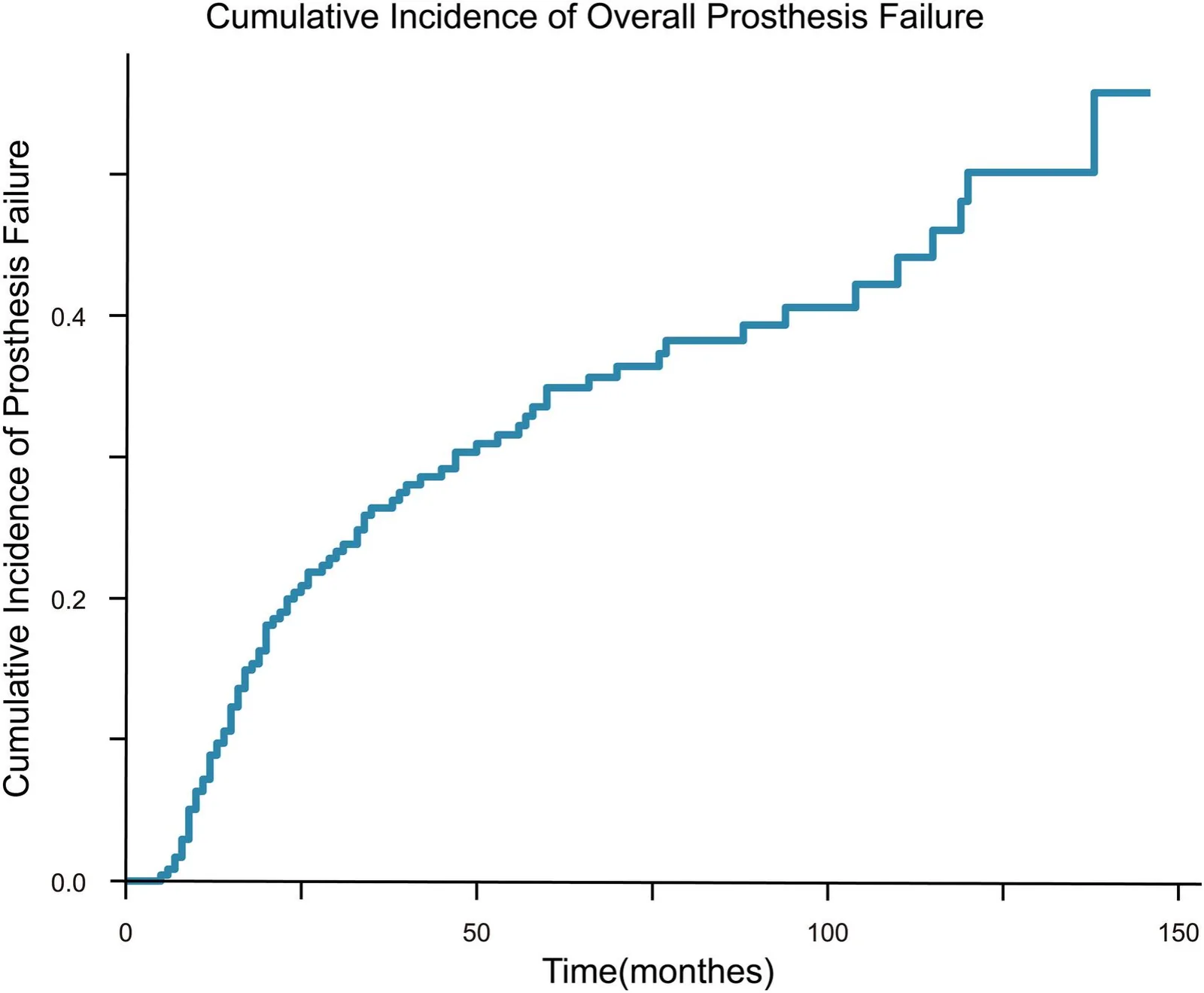
The cumulative incidence of overall prosthesis failure.

**Fig. 3:**
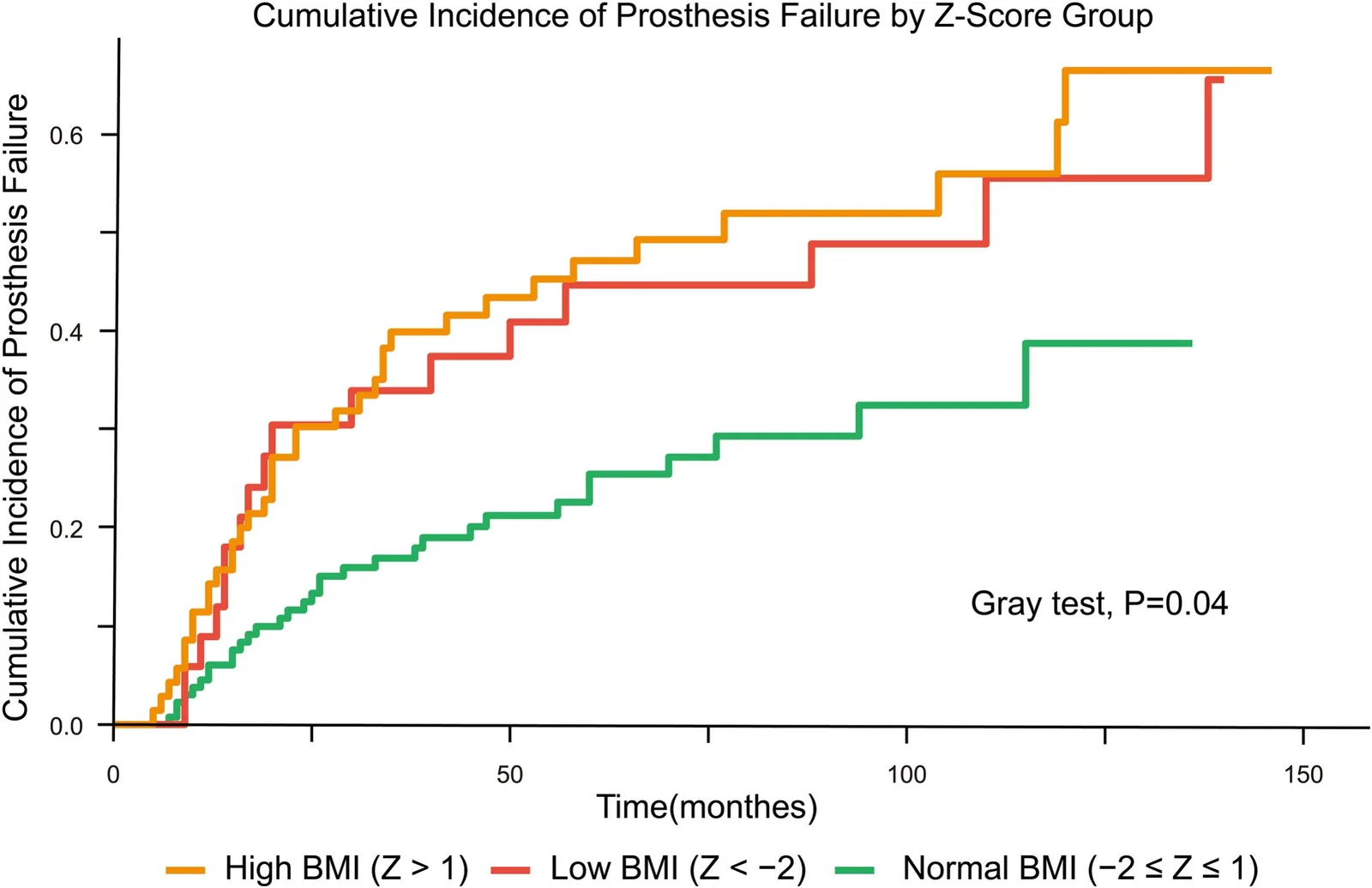
The cumulative incidence of Prosthesis Failure by Z-Score Group. BMI: Body mass index.

Restricted cubic spline analysis revealed a significant overall correlation between BMI *Z*-score and implant failure (*χ*^*2*^ = 14.53, df = 2, *P* = 0.001), with a nonlinear association (nonlinear *χ*^*2*^ = 5.23, df = 1, *P* = 0.022). The fitted curve was U-shaped, with turning points at Z = −2 and 1. Hazard ratios (HRs) rose significantly below −2. Between −2 and 1, HRs remained around 1, indicating a relatively safer nutritional range. Above 1, HRs increased gradually. This matched CIF results, where the normal-BMI group consistently had the lowest failure rate (Fig. 4).

**Fig. 4:**
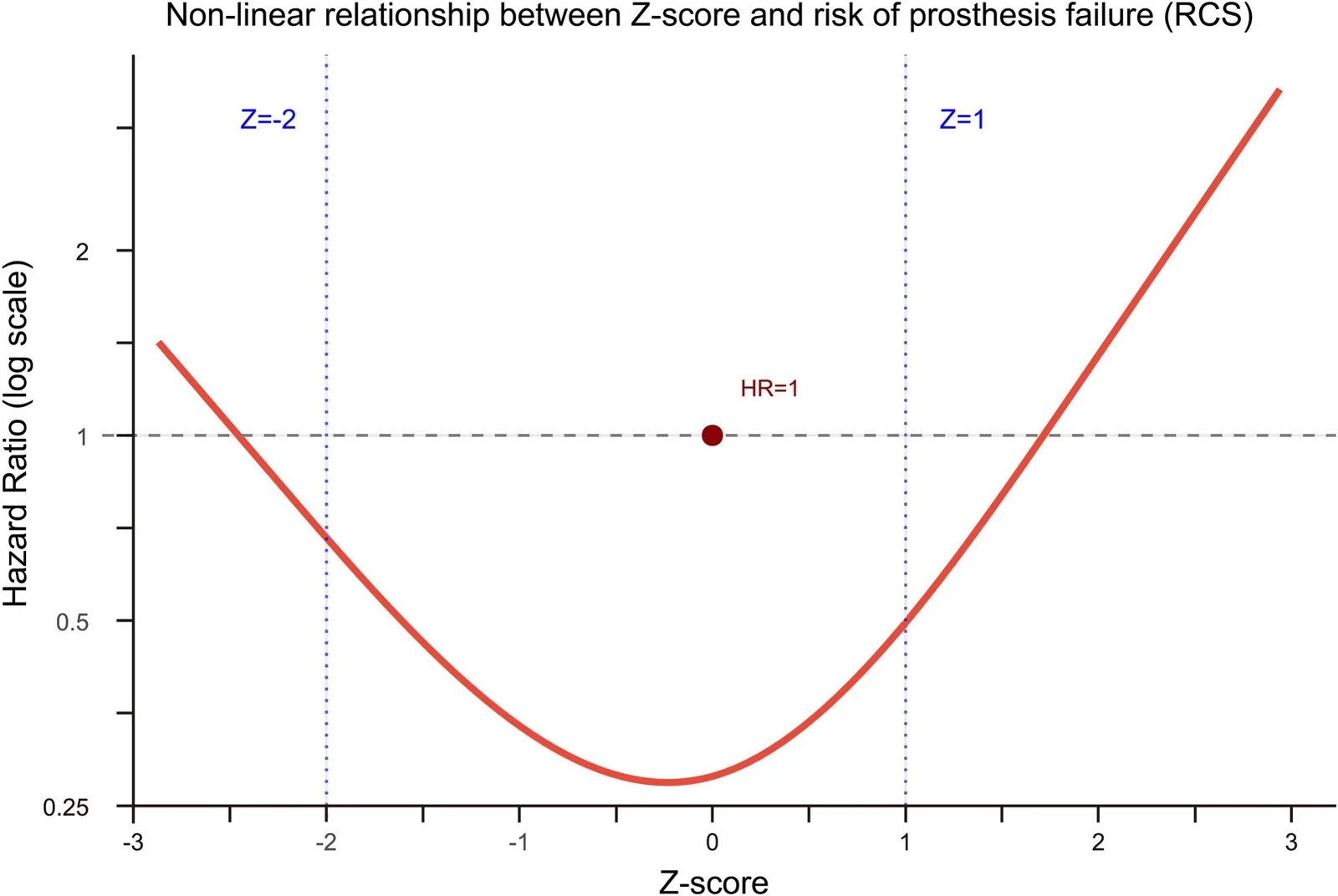
Non-linear relationship between Z-score and risk of prosthesis failure(RCS). RCS:Restricted Cubic Spline.

### 3.3 Risk factors for implant failure

Univariate Fine-Gray regression identified three independent risk factors for implant failure. Using normal BMI as the reference, low BMI (subdistribution hazard ratio [SHR] = 2.10, 95% CI: 1.18–3.76, *P* = 0.012) and high BMI (SHR = 2.28, 95% CI: 1.43–3.65, *P* = 0.001) were both associated with higher failure risk. Enneking stage III disease also carried greater risk than stage IIA (SHR = 2.31, 95% CI: 1.41–3.79, *P* < 0.001) Table 2. After adjustment for age, sex, tumor site, Enneking stage, and prosthesis type, BMI *Z*-score remained independently predictive: low BMI (SHR = 2.21, 95% CI: 1.20–4.05, *P* = 0.011) and high BMI (SHR = 2.26, 95% CI: 1.37–3.75, *P* = 0.002). In univariate analysis, age, sex, tumor location, and prosthesis type were non-significant. However, in the fully adjusted model, each additional year of age was linked to a 14.0% lower risk of failure (SHR = 0.86, 95% CI: 0.75–0.98, *P* = 0.024). Sex, tumor location, and prosthesis type remained non-significant Table 3.

**Table 2:** Univariable Fine-Gray competing risk analysis for risk factors of prosthesis failure.

Variable	Coefficient (β)	HR	95% CI	z value	*P* value
Age (years)	−0.05	0.96	0.89–1.02	−1.29	0.200
Sex (female/male)	0.32	1.38	0.91–2.10	1.49	0.140
Weight group (underweight/normal)	0.74	2.10	1.18–3.76	2.51	0.012
Weight group (overweight/normal)	0.83	2.28	1.43–3.65	3.45	0.001
Tumor site (1 vs. 2)	−0.22	0.80	0.25–2.60	−0.37	0.710
Tumor site (3 vs. 2)	0.30	1.35	0.70–2.62	0.90	0.370
Tumor site (4 vs. 2)	−0.05	0.95	0.42–2.15	−0.13	0.900
Enneking stage (II Avs. IIB)	−0.29	0.75	0.36–1.54	−0.78	0.430
Enneking stage (III vs. IIB)	0.84	2.31	1.41–3.79	3.31	<0.001
Prosthesis type (modular/extendable)	−0.18	0.84	0.52–1.36	−0.71	0.480

Tumor site: 1 = proximal humerus, 2 = proximal femur, 3 = distal femur, 4 = proximal tibia.

**Table 3:** Multivariable Fine-Gray competing risk analysis for risk factors of prosthesis failure.

Variable	Coefficient (β)	HR	95% CI	z value	P value
Age (years)	−0.15	0.86	0.75–0.98	−2.26	0.024
Sex (female/male)	0.33	1.40	0.90–2.15	1.50	0.130
Weight group (underweight/normal)	0.79	2.21	1.20–4.05	2.56	0.011
Weight group (overweight/normal)	0.82	2.26	1.37–3.75	3.17	0.002
Tumor site (1 vs. 2)	−0.10	0.90	0.28–2.88	−0.18	0.860
Tumor site (3 vs. 2)	0.34	1.41	0.77–2.57	1.12	0.260
Tumor site (4 vs. 2)	−0.06	0.94	0.42–2.12	−0.14	0.890
Enneking stage (II Avs. IIB)	−0.25	0.78	0.38–1.62	−0.66	0.510
Enneking stage (III vs. IIB)	0.79	2.19	1.35–3.56	3.18	0.002
Prosthesis type (modular/extendable)	0.70	2.01	0.78–5.16	1.45	0.150

Tumor site: 1 = proximal humerus, 2 = proximal femur, 3 = distal femur, 4 = proximal tibia.

### 3.4 Major postoperative complications

Major postoperative complications occurred in 109 patients (45.61%). The most common complications were soft tissue (11.30%), followed by PJI (10.88%), local tumor recurrence (10.04%), aseptic loosening (7.11%), and mechanical failure (6.28%). Complication rates differed significantly among BMI groups (*P* < 0.001): 61.8% in the low-BMI group, 31.9% in the normal-BMI group, and 64.3% in the high-BMI group Table 4.

**Table 4:** Postoperative Cumulative Incidence of Complications by BMI Z-Score Groups.

Complication	BMI Z-Score Group	Postoperative Cumulative Incidence (%)				Gray's test P value
		1 Year (12 Months)	3 Years (36 Months)	5 Years (60 Months)	10 Years (120 Months)	
Soft Tissue Complications	Low BMI (Z < −2)	15.69% (0.00–56.84)	19.61% (0.00–54.41)	19.61% (0.00–54.41)	19.61% (0.00–54.41)	0.430
	Normal BMI (−2 ≤ Z ≤ 1)	19.71% (0.00–64.72)	19.71% (0.00–64.72)	19.71% (0.00–64.72)	19.61% (0.00–58.69)	
	High BMI (Z > 1)	18.29% (0.00–62.03)	20.32% (0.00–55.59)	20.32% (0.00–55.59)	20.32% (0.00–55.59)	
Aseptic Loosening	Low BMI (Z < −2)	0.00% (0.00–0.00)	3.92% (0.00–25.89)	11.76% (0.00–35.63)	15.69% (0.00–39.45)	0.274
	Normal BMI (−2 ≤ Z ≤ 1)	0.00% (0.00–0.00)	1.79% (0.00–16.80)	7.17% (0.00–24.02)	10.75% (0.00–26.97)	
	High BMI (Z > 1)	0.00% (0.00–0.00)	2.03% (0.00–18.00)	6.10% (0.00–23.82)	12.19% (0.00–29.98)	
Mechanical Failure	Low BMI (Z < −2)	0.00% (0.00–0.00)	0.00% (0.00–0.00)	0.00% (0.00–0.00)	3.92% (0.00–25.89)	0.308
	Normal BMI (−2 ≤ Z ≤ 1)	0.00% (0.00–0.00)	3.58% (0.00–19.88)	8.96% (0.00–25.83)	12.54% (0.00–28.77)	
	High BMI (Z > 1)	0.00% (0.00–0.00)	6.10% (0.00–23.82)	10.16% (0.00–28.01)	14.22% (0.00–31.90)	
Periprosthetic Infection	Low BMI (Z < −2)	19.61% (0.00–45.55)	23.53% (0.00–48.60)	23.53% (0.00–48.60)	23.53% (0.00–48.60)	0.012
	Normal BMI (−2 ≤ Z ≤ 1)	10.75% (0.00–30.99)	12.54% (0.00–32.11)	12.54% (0.00–32.11)	12.54% (0.00–31.28)	
	High BMI (Z > 1)	24.38% (3.97–44.79)	26.41% (6.59–46.24)	26.41% (6.59–46.24)	26.41% (6.59–46.24)	
Local Recurrence	Low BMI (Z < −2)	3.92% (0.00–25.89)	15.69% (0.00–39.45)	15.69% (0.00–39.45)	15.69% (0.00–39.45)	0.681
	Normal BMI (−2 ≤ Z ≤ 1)	8.96% (0.00–30.11)	21.50% (1.97–41.03)	21.50% (1.97–41.03)	21.50% (2.52–40.48)	
	High BMI (Z > 1)	6.10% (0.00–23.82)	16.25% (0.00–36.31)	16.25% (0.00–36.31)	16.25% (0.00–36.31)	

Data are presented as cumulative incidence (%) (95% CI).

### 3.5 Competing risk analysis of complications

Soft tissue complications clustered shortly after surgery, and all CIF curves plateaued after 3 years. By then, cumulative incidence was 19.61% (95% CI: 0.00%–54.41%) in the low-BMI group, 19.71% (95% CI: 0.00%–64.72%) in the normal-BMI group, and 20.32% (95% CI: 0.00%–55.59%) in the high-BMI group. The between-group difference was minor and non-significant (Gray test, *P* = 0.430) Table 4.

Aseptic loosening followed the typical late-complication pattern, with risk gradually increasing over time. At 10 years, cumulative incidence was highest in the low-BMI group at 15.69% (95% CI: 0.00%–39.45%), followed by the high-BMI group at 12.19% (95% CI: 0.00%–29.98%) and the normal-BMI group at 10.75% (95% CI: 0.00%–26.97%). The differences were non-significant (Gray's test, *P* = 0.274) Table 4.

Mechanical failure also revealed a time-related pattern. At 10 years, cumulative incidence was 3.92% (95% CI: 0.00%–25.89%) in the low-BMI group, 12.54% (95% CI: 0.00%–28.77%) in the normal-BMI group, and 14.22% (95% CI: 0.00%–31.90%) in the high-BMI group. Again, the differences were non-significant (Gray's test, *P* = 0.308) Table 4.

PJI was observed mostly in the early postoperative period, with no additional rise in cumulative incidence after 3 years. At that point, the normal-BMI group had the lowest cumulative incidence at 12.54% (95% CI: 0.00%–32.11%), whereas rates were higher in the low-BMI group at 23.53% (95% CI: 0.00%–48.60%) and the high-BMI group at 26.41% (95% CI: 6.59%–46.24%) (Gray's test, *P* = 0.012) Table 4.

Local recurrence increased significantly between postoperative years 1 and 3, then plateaued. The 10-year cumulative incidence was 15.69% (95% CI: 0.00%–39.45%) in the low-BMI group, 21.50% (95% CI: 2.52%–40.48%) in the normal-BMI group, and 16.25% (95% CI: 0.00%–36.31%) in the high-BMI group. Differences in BMI strata were non-significant (Gray's test, *P* = 0.681) Table 4.

## 4 Discussion

Abnormal weight status, as defined by BMI-for-age, was associated with poor endoprosthetic outcomes in pediatric osteosarcoma. Both underweight and overweight patients exhibited approximately a two-fold increased risk of implant failure, as well as more complications. Restricted cubic spline analysis demonstrated a significant U-shaped relationship between BMI *Z*-score and the risk of implant failure (nonlinear *P* = 0.022; overall association *P* = 0.001), indicating that the lowest risk occurs within the normal-weight range (−2 ≤ Z ≤ 1). A similar pattern was observed in sensitivity analyses after multivariable adjustment, including a competing-risks model that accounts for mortality. In essence, the BMI-for-age *Z*-score may be a clinically meaningful and partially modifiable host factor in pediatric limb-salvage reconstruction.

These findings expand our current understanding of risk in arthroplasty and oncologic reconstruction. Prior reports have primarily focused on obesity as a cause of infection, wound problems, and mechanical failure [4], [13], while the adverse effect of low BMI has generally been disregarded [14], [15]. Earlier research also tended to treat BMI as a monotonic linear variable or use adult cutoffs, neither of which accurately represented the anthropometric reality of pediatric patients [16], [17]. Children differ from adults in terms of skeletal maturity, growth rate, chemotherapy exposure, soft-tissue reserve, and functional demand [16]. Using age- and sex-standardized BMI-for-age Z-scores with nonlinear modeling, our data reveal that the association between body habitus and implant outcome is not monotonic, and BMI alone does not fully explain adverse risk after pediatric limb salvage.

The association at the lower end of the BMI spectrum is both biologically plausible and clinically relevant [18], [19]. A low BMI in patients with pediatric osteosarcoma receiving multimodal treatment may indicate poor nutritional reserve, impaired immunity, and decreased soft-tissue repair capacity [20], [21], [22]. This may increase wound complications and infections as well as impaired osseous integration, increasing the risk of aseptic loosening [23], [24]. In patients with a low BMI, altered drug metabolism during treatment may increase chemotherapeutic toxicity and adversely affect overall survival [25]. In our cohort, the low-BMI group had lower implant survival rates and more complications, indicating that undernutrition is a significant host-related vulnerability rather than a neutral baseline trait. Consequently, low BMI should be interpreted as a signal for intensified perioperative management, rather than a descriptive variable.

The negative association at the higher BMI levels also fits plausible clinical mechanisms. Increased body mass may increase stress at the bone-implant interface and impair soft-tissue healing after oncologic resection, leading to loosening, structural failure, wound complications, and infection [26], [27]. Chemotherapy-damaged tissues and fixation demands in pediatric reconstruction may amplify the impact of excess weight [28], [29]. Excess body weight may also aggravate chemotherapeutic toxicity and reduce overall survival [30]. Our study does not establish a causal link between high BMI and implant failure, but it does identify patients with the highest BMI-for-age levels as a high-risk subgroup that requires counseling and follow-up.

Restricted cubic spline modeling also revealed a significant U-shaped relationship between BMI-for-age *Z*-score and implant failure (nonlinear *P* = 0.022; overall association *P* = 0.001), with the lowest risk in the normal nutritional range. In other words, implant survival is unlikely to worsen in a single direction; deviation on either side of an age- and sex-appropriate range may harm the outcome through different mechanisms [31], [32]. This is consistent with arthroplasty literature linking obesity to inferior implant outcomes as well as data indicating that low BMI may reflect reduced physiologic reserve and increased vulnerability to complications [33], [34]. Given the limited evidence in pediatric and adolescent osteosarcoma reconstruction, growth-adjusted BMI Z-score modeling provides a more nuanced prognosis than dichotomous or linear BMI assessment.

The complication profile also supports the clinical significance of abnormal BMI. Periprosthetic infection appeared to be the most common adverse event at both BMI extremes, while aseptic loosening and mechanical failure were also more frequent in patients with abnormal BMI [35], [36]. Local recurrence was included in the overall failure spectrum because, in limb salvage, it can directly threaten implant retention, resulting in revision, removal, or amputation [29], [37]. Although biologically distinct from mechanical and infectious failure, it is an oncologic event that has a secondary impact on implant survival. This framework more accurately reflects the actual overlap between oncologic and reconstructive failure in pediatric limb salvage.

Several methodological points contribute to the reliability of our findings. The consecutive single-center design decreased variation in indications, reconstructive strategy, and follow-up. BMI was calculated using WHO pediatric growth references, which are a more age-appropriate metric than adult BMI categories [38], [39]. We examined both implant survival and postoperative complications to get a broader view of clinical burden [1], [40]. Sensitivity analysis limited to lower-extremity reconstruction yielded directionally and statistically consistent results. Additionally, competing-risk modeling with death as a competing event addressed a critical issue in oncologic cohorts that are frequently missed by standard survival methods [41], [42].

This study has several limitations. Despite multivariable adjustment, its retrospective single-center design allows for selection bias and unmeasured confounding. The relatively small number of failure events may reduce the stability of some adjusted estimates, particularly with multiple covariates or subgroup analyses. BMI-for-age was recorded once at diagnosis, before the start of neoadjuvant chemotherapy. This single time point does not capture weight change during chemotherapy or during the postoperative course, and it represents the least modifiable point in the treatment pathway. It is therefore best interpreted as a baseline marker for preoperative risk stratification rather than as evidence that weight modification would alter implant survival. Longitudinal studies that measure BMI and body composition at several time points during and after treatment are needed to determine whether weight trajectory influences prosthetic survival. Additionally, laboratory nutritional markers, inflammatory indices, and body composition data were unavailable, limiting our ability to more comprehensively assess host reserve. The inclusion of local recurrence in the overall failure definition was clinically justified but inevitably introduced endpoint heterogeneity.

## 5 Conclusions

Abnormal BMI-for-age, at both low and high levels, was associated with increased implant failure risk and a higher complication burden after endoprosthetic reconstruction in pediatric osteosarcoma. The U-shaped pattern suggests that deviation from the normal BMI-for-age range in either direction—rather than just high BMI—could affect implant survival. If confirmed in larger multicenter cohorts, BMI-for-age could be a simple preoperative risk-stratification tool for counseling and postoperative surveillance.

## CRediT authorship contribution statement

**Song Peng:** Writing – original draft, Methodology, Investigation, Formal analysis, Data curation, Conceptualization. **Zhilong She:** Writing – original draft, Investigation. **Jiahao Chen:** Validation, Investigation, Data curation. **Qiong Wu:** Validation, Formal analysis, Data curation. **Guangxin Zhou:** Writing – review & editing, Validation, Supervision. **Yicun Wang:** Writing – review & editing, Validation, Supervision, Conceptualization.

## Declaration of generative AI and AI-assisted technologies in the writing process

During the preparation of this manuscript, the authors used ChatGPT-4 (OpenAI) for language polishing, grammar correction, and phrasing refinement only. The AI tool was not used to generate core scientific content, including study design, data analysis, result interpretation, or clinical conclusions. All content was reviewed, edited, and finalized by the authors under strict human oversight. The authors take full and final responsibility for the accuracy, integrity, and originality of the entire manuscript.

## Declaration of competing interest

The authors declare that they have no known competing financial interests or personal relationships that could have appeared to influence the work reported in this paper.
